# The relevance of pre-exposure prophylaxis in gay men’s lives and their motivations to use it: a qualitative study

**DOI:** 10.1186/s12889-021-11863-w

**Published:** 2021-10-09

**Authors:** Jorge L. Alcantar Heredia, Shelly Goldklank

**Affiliations:** Adult Clinical PsyD Program, Ferkauf Graduate School of Psychology, Yeshiva University, 1165 Morris Park Ave., The Bronx, NY 10003 New York, USA

**Keywords:** PrEP, Gay, GMSM, Relevance, Uptake

## Abstract

**Background:**

HIV has affected gay men disproportionately in the U.S. for four decades. Pre-exposure prophylaxis (PrEP) was developed as a prevention strategy for individuals at high risk of HIV infection. Although highly effective, many gay and other men who have sex with men continue not to take PrEP. Researchers have focused on sexual risk behaviors as the primary determinant of who should be on PrEP and identified various objective systemic and societal barriers to PrEP access. Public health measures have promoted PrEP based on these objective criteria. Researchers have recently begun to inquire into subjective and relational motivators for PrEP usage beyond self-perceived risk.

**Methods:**

Participants were recruited through snowball sampling. Data were collected between August and November 2018 from PrEP users (*n* = 7) and PrEP non-users (*n* = 6). Data were analyzed in a modified grounded theory qualitative analysis.

**Results:**

The thirteen participants’ narratives contained three superordinate categories: (1) what it’s like to be someone on PrEP, (2) an environment of changing sexual norms, and (3) the continued importance of education. These categories comprised ten themes, each of which had various repeating ideas. The ten themes were the following: (1) PrEP’s social acceptability, (2) PrEP and HIV stigma, (3) PrEP and sexual relationships, (4) dissatisfaction with condoms, (5) negotiating risk, (6) peace of mind, (7) developing a relationship with PrEP, (8) putting yourself first, (9) PrEP awareness, and (10) PrEP logistics.

**Conclusions:**

The gay men in our study took into consideration their social roles and relationships, their personal beliefs, and emotional histories as well as risk as prominent motivators for PrEP use. They stated that PrEP use is associated with their sense of belonging, trust, and security about their sexuality. They also identified the most relevant aspects of the medication (e.g., side effects, adherence, and awareness) to their lives.

**Supplementary Information:**

The online version contains supplementary material available at 10.1186/s12889-021-11863-w.

## Background

In 2012, the U.S. Food and Drug Administration (FDA) approved a new human immunodeficiency virus (HIV) prevention strategy, pre-exposure prophylaxis (PrEP) [1]. Although highly effective, PrEP has not been highly accessed by many populations at high risk of HIV infection including gay and other men who have sex with men (collectively GMSM) [2]. In the U.S., HIV infection rates among GMSM remain higher than those of the rest of the population [3].

Three prominent areas of PrEP research have focused on PrEP’s medical utility (e.g., side effects and effectiveness), its impact on behaviors such as *risk compensation* (e.g., how individuals adjust their behavior in relation to their level of perceived risk), and its rates of utilization and uptake. Data consistently show that PrEP is highly effective [4–6] and that side effects are relatively minor, absent for most, and usually resolve in the first 3 months of treatment [7].

Data on sexual risk compensation, on the other hand, have been less straightforward. A recent review noted that previous findings indicating no evidence of risk compensation among PrEP users relied on data from blind trials in which participants were not certain whether they were receiving PrEP [8]. Data from real-world settings, however, indicate that sexually transmitted infection (STI) diagnoses, condomless anal sex (CAS), and other sexual risk behaviors, are higher among PrEP users [8]. Many researchers contend that the reduction in risk of HIV infection from PrEP use exceeds the increased risk from behavioral changes [9].

PrEP uptake among some populations at greatest risk of HIV infection, such as young GMSM and GMSM of color, remains low [2]. Research has focused on describing how PrEP is utilized by GMSM, including what facilitates and hinders PrEP use, hoping to develop interventions that increase uptake [10] and persistence [11–16]. This research indicates PrEP uptake is significantly affected by awareness of it and its acceptability, cost and healthcare barriers, and PrEP-related stigma [17, 18].

As early as 2015, researchers began to theorize how the various individual and interactional meanings behind sexual behaviors and intimacy and partnership dynamics might affect PrEP use [19, 20]. They suggested that further research into these domains held the potential to orient clinicians in their work with GMSM individuals or couples who are considering whether to use PrEP [19, 20]. Subsequent research has suggested that, for GMSM who use PrEP, relational factors, including intimacy, and pleasure influence engagement in sexual risk behaviors and PrEP uptake [21–23]. Other studies have suggested that HIV-negative GMSM in serodiscordant partnerships might be interested in adopting PrEP in the future to decrease their anxiety about engaging in CAS with a serodiscordant partner, for HIV protection, and to engage in sex using a noncondom HIV prevention method [24].

Ethnographic studies about PrEP use and research about the subjective and relational factors associated with PrEP use have produced valuable findings while remaining much less developed than research on PrEP’s utility, behavioral effects, and utilization [25]. There is also a dearth of research that explicitly addresses how partnered consensual non-monogamy (PCNM) affects PrEP and its absence from public health messaging [26]. We hope our study enriches extant PrEP research through a grounded theory approach [27] via interviews of gay men who have considered using PrEP, focusing on the beliefs, feelings, and experiences that undergird their sense of the relevance of PrEP use to their lives that then affect whether they are motivated to use it.

## Methods

The current study used a semi-structured qualitative interview so that participants could explain their thoughts, feelings and actions and the consequences of those phenomena freely rather than being confined to pre-formulated constructs of a questionnaire or a quantitative measure. Eligible participants were recruited by the researchers through emails on their personal and professional networks, through flyers at local LGBT service centers, and through Reddit message boards “askgaybros,” “gay,” “SampleSize,” “hivaids,” and “PrEPared.” All participants were males who self-identified as gay, English- or Spanish-speaking, over age 18, and sexually active within the past 6 months. Additionally, participants must have heard of Truvada for PrEP and considered using it; those who used PrEP and those who did not use PrEP were encouraged to participate. The researchers and the participants set up a time to meet for the interview. Demographic data, including whether the men were sexually active in the last 6 months, were collected at screening through a questionnaire tailored to this study (Additional file 1, Demographic Questionnaire). This data collection instrument was developed specifically for this study. Items selected as typical demographic data used in PrEP studies were sex, gender, age, ethnicity/race, highest level of education, occupation, and estimated annual income (e.g., [28]). The questionnaire also included items tailored to this study: HIV status and whether the participant was sexually active in the past 6 months, each participant’s current relationship status, including how long he had been in a relationship, whether the relationship was monogamous, and the partner’s HIV status. “Sexually active” was left undefined. The researchers also developed a semi-structured interview (Additional file 2, Semi-structured Interview). This data collection instrument was developed specifically for this study. The prompts for this interview were developed based on the extant literature as foci of concern (e.g., [28]). The researchers then discussed the inclusion of these items and agreed on their relevance and wording. The researchers did not pilot test these items. Not all prompts were used with all participants. The researchers added additional prompts as new ideas and concerns were raised by participants over the course of the interview process. These additional prompts were asked in as neutral a manner as possible so as not to lead the participant. The semi-structured interview potential prompts that were particular to the aims and goals of this study and included items such as, ‘How did you learn about PrEP? What was going on in your life when you decided to take PrEP, and how did you decide whether to take it? How do you think being on PrEP has affected you? Does being on PrEP affect your willingness to be in a committed relationship? Does being on PrEP affect your willingness to be in an open relationship? Have you considered using PrEP? Are you currently on PrEP? How often do you take your PrEP medication? Have you experienced side effects while taking PrEP? What is your HIV status? When were you last tested? The researchers then discussed the inclusion of these items and agreed on their relevance and wording. The researchers did not pilot test these items. They did, however, add additional prompts as ideas began to repeat across participants over the course of the interview process. Participants met in person with the researchers at locations where privacy could be maintained (e.g., libraries, parks, private homes). Participants were not compensated. The researchers addressed concerns about confidentiality and any other concerns the men might have upon meeting, and provided participants with written informed consent forms. At the outset of every interview, participants were given the prompt, “We’re interested in knowing your thoughts and feelings about PrEP, and whether you use it or you don’t use it. We’re interested in the relationship of its use (or not) to your intimate sexual relationships and sex practices. Could you please tell us?” Additional prompts were used to explore participants’ experience further. As mentioned above, a set of potential prompts was created at the outset of the study and grew over the course of the study. Not every prompt was asked of each participant. In concluding, the men in the study were asked if they had anything to add and if they wanted to hear about the study’s progress. Audio recordings were subsequently transcribed and de-identified. Each participant was given a code name based on his PrEP use status. The Institutional Review Board of the Albert Einstein College of Medicine at Yeshiva University, New York, NY, approved the study (IRB# 2016–6444).

### Data analysis

Preliminary data analysis consisted of a descriptive analysis of the sample (e.g., average age, age range, racial/ethnic composition, average level of education of the sample).

We analyzed the deidentified interview data using a modified version of Auerbach and Silverstein’s qualitative method [29]. We used the steps identified by these authors to identify relevant text and to discover repeating ideas (RIs), i.e., “an idea expressed in relevant text by two or more research participants” [29], by grouping together related passages of relevant text. We organized common themes by grouping RIs into coherent units. We used a stringent rating process wherein four raters had to reach consensus about whether ideas were repeated, before we then reached consensus about what groups of RIs fit together into themes. Then, by organizing themes into more abstract concepts, we developed superordinate categories informed by a theory in the extant literature or novel constructs that related to those data. Our modifications were: particularizing participants by providing deidentified narratives for each, abstaining from generalizing our findings, and applying aspects of Clara Hill et al.’s [30] Consensual Qualitative Research by interpreting the data and reaching consensus among four raters and disclosing biases for each member to mitigate their effect.

## Results

### Demographic data

Thirteen research participants, in their 20s and 30s, enrolled between August and November 2018. Tables 1a and 1b include demographic data for these participants (see Additional file 3, Tables 1a and 1b). All participants were cisgender males. Interviews lasted from 22 to 62 mins. In one instance, a participant expressed concern about his voice being recorded; thus, his responses were typed. Participants were closely split between PrEP users and PrEP non-users. Seven participants reported using PrEP for a range of two months to five years (M = 2.8 years). Seven participants reported being single (5 PrEP users and 2 PrEP non-users), and six participants reported being in a committed relationship (2 PrEP users and 4 PrEP non-users). Participants’ committed relationships (*n* = 6) ranged from 1.5 to 16 years (M = 4.6 years). Twelve individuals (92%) identified as Caucasian/White. Nine individuals (69%) reported some form of higher education; the remaining four preferred not to disclose their level of education. The average yearly income of this sample was approximately $60,000. Thus, this project’s sample comprised a largely homogenous group of Caucasian, highly educated, middle to upper-middle class, cis gay men, seven single and six in some form of committed partnership.

#### Narratives of participants targeting the relevance and motivations of PrEP use

In the following narratives, participants are identified by whether they use PrEP (e.g., U1 is the first participant, and he uses PrEP), or do not use PrEP (e.g., N4, the fourth participant in the study). Their other identifying data were translated into generics to protect confidentiality.

U1 (in his 30s) does not remember whether he found out about PrEP through his doctor or if he just heard about it. When he started PrEP, he was interested in going on it because he recognized that he engaged in CAS and wanted additional protection.

U2 (in his 30s) heard about PrEP from a friend of a friend but was incredulous that such a pill could exist. He did not use PrEP for a year due to concerns about its novelty and possible side effects. U2 decided to start PrEP when he became less concerned about side effects; he also observed that CAS was increasingly prevalent and became more concerned about the possibility of HIV infection.

U3 (in his 20s) has been in an open relationship for 2 years with an HIV-negative gay man who is also on PrEP. U3 started to take PrEP after he heard about it through his Primary Care Physician at an LGBT-friendly clinic, who told our participant that he was at high risk of HIV infection due to his regular engagement in CAS.

N4 (in his 20s) has been in an open relationship for 1.5 years with an HIV-negative gay man. N4 perceives his risk of HIV infection to be low and is not currently on PrEP. He is primarily sexually active with his romantic partner, with whom he has CAS, and they “play together” with extra dyadic partners with whom they use condoms. N4 used PrEP prior to this relationship. This couple was beginning to consider whether to incorporate PrEP into their sexual contract.

N5 (in his 30s) used PrEP for 3 years but stopped 1.5 years ago when he entered a sexually monogamous bond with an HIV negative gay male partner who is not on PrEP. They recently agreed to include extradyadic sexual partners with whom they use condoms in their relationship. They are considering starting PrEP but believe they do not “play with others” often enough to offset the “costs” of using PrEP (e.g., going to the doctor, taking a daily medication).

U6 (in his 30s) is in a sexually non-monogamous marriage with an HIV-negative gay man who also uses PrEP. They have been together for 5 years. U6 heard about PrEP through his husband. At first, U6 feared using PrEP might introduce power inequality in their relationship. However, he grew to believe that using PrEP would primarily increase his and his partner’s safety. U6 and his husband have sex with extradyadic partners separately as well as together and are both flexible with their condom use.

U7 (in his 30s) had wanted to use PrEP to reduce his risk of HIV infection and his fear of HIV for some time, but started only after an HIV scare.

U8 (in his 20s) came across PrEP in 2015 when he read about it in an American blog. He started PrEP about 2 years later as he saw friends with similar sexual behaviors starting PrEP.

U9 (in his 30s) found out about PrEP through reading in 2014, and, though he immediately bought into PrEP, it was not until a friend was diagnosed with HIV several years later that he decided to start taking Truvada.

N10 (in his 20s) first heard about PrEP through his university’s health center while getting an STI test. He is not currently on PrEP because of concerns about side effects and because he believes his risk to be low given his regular condom use. Nonetheless, he is considering using PrEP as additional protection against HIV.

N11 (in his 20s) is not on PrEP because he does not believe himself to be at elevated risk of HIV infection and because he is concerned about side effects and the high cost of being on PrEP. Because his friends use it, he has wondered whether PrEP might be relevant to him to further reduce his risk of contracting HIV.

N12 (in his 30s) is sexually active exclusively with his romantic partner, an HIV-negative gay man, within the context of a sexually monogamous relationship. They were in a committed relationship for 20 months at the time of the interview. N12 learned about PrEP through social media. He regards PrEP as one of several useful tools to prevent HIV. N12 considered using PrEP before committing to his current relationship because he occasionally had casual sexual encounters.

N13 (in his 30s) has been in a sexually monogamous relationship for 16 years with his now husband. He learned about PrEP through an LGBT organization. N13 regularly engages in extramarital sexual encounters involving methamphetamine use with numerous sexual partners. He perceives his risk of HIV infection to be high and plans to start PrEP to protect his and his husband’s health after finishing his current round of post-exposure prophylaxis.

### Categories, themes, and repeating ideas

Three superordinate categories, comprising 10 themes, emerged from our analysis. These results are summarized in a table containing the categories, themes, and repeating ideas that emerged (see Additional file 4, Table 2). The first category, what it’s like to be someone on PrEP, included the themes (1) belongingness and social acceptability, (2) fear and stigma associated with sex, and (3) trust and sexual relationships. The second category, an environment of changing sexual norms, included the themes (4) dissatisfaction with condoms, (5) negotiating risk, (6) peace of mind, (7) developing a relationship with PrEP, and (8) putting yourself first. The third category, the continued importance of education, included the themes (9) PrEP awareness and (10) PrEP logistics.

### Overview

#### Category 1: What’s It’s like to be someone Who’s on PrEP

For the men in our study, deciding whether to use PrEP involved considering the relevance and meaning of using PrEP in different personal and social contexts. Their experience led to discussion of the themes of belongingness and social acceptability, fear and stigma associated with sex, and trust within partnerships.

The men in our study often talked about how being on PrEP affected their sense of belongingness and social acceptability within their social group. How their friends, the friends of their friends, and their lovers talked about PrEP affected whether our participants believed PrEP to be relevant for them. U2 (30s, single, $35 K) seemed to feel he was part of this group and stated, “Being surrounded by people who take PrEP, and there’s nothing wrong with them. They’re in perfect health. It doesn’t affect them negatively.” N10 (20s, single, no income declared), a PrEP non-user, stated, “I’m not even sure if any of my closest friends are on PrEP. It’s not something I’ve really discussed with friends or people around me. I don’t know if some of my friends were like, ‘Oh, I’m on PrEP and it’s amazing,’ if that would make me more likely to, but right now there’s not any active pressure or opinion in either direction in my closest friend group.” Several participants (*n* = 5) mentioned that general social acceptability affected their interest in PrEP, and that they felt pressured to be on PrEP. Moreover, participants (*n* = 4) suggested that being on PrEP was relevant not just for them personally but also for the larger community. U7 (30s, single, $140 K) stated, “It’s sort of important for most, I feel there’s sort of a political thing, I think it’s important for anyone who can go on [PrEP] to go on it because it will radically decrease the number of new HIV infections in the United States.” U7 also stated, “The perception of PrEP is largely positive. But. .. some people’s perception of PrEP is colored by their general perception. .. of sex.” By drawing the connection between attitudes towards PrEP and attitudes towards gay sex, U7 explicitly acknowledged that PrEP use carries social meaning and represents more than just an HIV prevention strategy.

For the men in our study, the meaning of identifying as using PrEP extended beyond notions of belonging and community and was related to fear and stigma associated with sex and sexual identity. Nearly all participants (*n* = 12) associated PrEP use and stigma; they expressed both that stigma affected what they thought about using PrEP, and that being on PrEP shifted their own internalized stigmatizing biases. Participants (*n* = 7) stated that using PrEP is associated with promiscuity and risky behavior and that PrEP stigma leads to shame and judgment of gay men who use PrEP. U1 (30s, single, $10 K) shared, “People shame people for certain sexual activities. .. If you’re going on PrEP, you must be a slut. .. People are getting devalued for using PrEP because they’re just pieces of ass or just people to fuck.” U1 associated these views about PrEP specifically with “bottoms” (i.e., men who engage in anal receptive sex) and, “being a top,” he did not comment further on their relevance to him. N13 (30s, closed relationship, $12 K), who previously used PrEP but no longer did at the time of this study, described being wary of disclosing his PrEP use to his friends. “It’s not something I would tell anybody. .. because to say something to even a friend it sort of becomes, like, why are you on it? You’re married. Usually, it’s somebody who’s not married who says that. And it’s definitely not something I’d want somebody to know because of the picture of what my marriage is.” Participants (*n* = 6) also observed a decrease in HIV stigma as a result of PrEP. U4 stated, “I think that, now PrEP has lightened the tone. .. I feel more comfortable talking about HIV/AIDS because people aren’t as at risk as they were before PrEP.”

Our participants discussed how being on PrEP was relevant to their sense of trust and their sexual relationships and how PrEP use affects how they show up in relationships, as primary partners in committed relationships or as sex partners in casual encounters. They repeatedly addressed the ideas of trust and security. Most participants (*n* = 12) expressed that PrEP use shapes attitudes towards casual sexual encounters and committed partnerships. Some (*n* = 6) felt more comfortable having sex with casual sexual partners they trusted less. Many participants (*n* = 5) stated that PrEP use feels relevant especially for men in open relationships.

The question of whether PrEP is relevant for men in monogamous relationship agreements was widely addressed (*n* = 11) and raised a range of views. While some participants supported the use of PrEP within monogamous relationship for peace of mind, sexual health agency, and to protect the dyad’s sexual health, some PrEP non-users stated that they stopped using PrEP upon entering their current relationships because both partners agreed to be monogamous. Some stated that they trusted each other not to breach that agreement, while others avoided bringing up starting PrEP for fear of seeming distrustful or eliciting distrust. Most PrEP non-users and individuals who were single and on PrEP had preexisting views about PrEP use in monogamous relationships. N10 (20s, single, no income declared), speculated, “I feel that if I was in a long-term relationship, especially a monogamous one, I would feel more inclined not to use condoms, and in that scenario maybe I would want, if I want to be extra safe, maybe I would want to get on PrEP in that situation because we’re not using condoms.”

Some participants (*n* = 6) spoke about how PrEP use affects their sense of trust with casual and committed sexual partners. Specifically, PrEP-using and PrEP non-using participants alike talked about how PrEP has been useful because they are able to have sex with partners, whom they are not fully able to trust, without worrying whether their partners are being truthful. U8 (20s, single, $60 k) stated, “It completely eliminates your need to take someone’s word. If they say they’re on PrEP, I don’t have to worry about them telling you the truth or not. .. I don’t have to worry about your status, basically, and you being honest about the partners you’ve had.”

Both PrEP non-users and PrEP users (*n* = 5) described how using PrEP either might affect or had affected being in an open relationship. They associated PrEP use with less worry for themselves and for their partners and with increased trust from feeling that they and their partners were protecting each other’s health. U6 (30s, open marriage, $19 k) stated, “We’re both enjoying extramarital experiences. In a way, that also makes me trust him more as well. I mean, about being safe and not getting HIV. .. protect yourself and protect the ones you love.”

Four participants discussed whether PrEP had some bearing on their interest in being in committed relationships. Two thought PrEP had no impact on their interest in committed relationships, and two believed PrEP use decreased interest in being in a committed relationship because it allowed them to have CAS outside of sexually monogamous committed relationships. N5 (30s, open relationship, $86 k) stated, “One of the reasons why people used to say they liked being in a monogamous relationship was because then you can have bareback sex and that feels better. .. PrEP sort of took that out of the picture. .. not that some people are not willing to be in a committed relationship. It’s just that they maybe don’t feel quite the urgency to be in it.”

#### Category 2: an environment of changing sexual norms

When the men in our study decided whether PrEP was right for them, they considered PrEP’s relevance to them in an environment of changing sexual norms, in which some PrEP use was directly responsible for this change. This category comprised five themes that clarify how PrEP is related to attitudes about distinct aspects of this changing sexual environment: dissatisfaction with condoms, negotiating risk, peace of mind, developing a relationship with PrEP, and putting yourself first.

Many participants (*n* = 11) expressed a sense of dissatisfaction with condoms. They described various ways in which this dissatisfaction was related to their interest in using PrEP or to their sense of PrEP as being relevant to them. Participants associated condoms with sex that was less pleasurable, less spontaneous, and less safe. Although participants described enjoying barebacking more than sex with a condom, no participant expressed that his decision to use PrEP was motivated by a desire to bareback more. Three participants stated that PrEP use was relevant to them because they were concerned about whether condoms reliably protected them against HIV. U6 (30s, open marriage, $19 k) feared that a condom “could get broken, that’s a piece of plastic; PrEP is something that’s in you.” Participants expressed a desire for sex to be spontaneous, which PrEP facilitated. N5 (30s, open relationship, $86 k) stated, “You can just [have sex]. .. you can have sex at the club, you can have sex at the sex party, you can do all of these things and you’re not having to bring condoms.”

Dissatisfaction with condoms was evident within and relevant to the wider social shift away from condom use for our participants. Five participants, both PrEP users and PrEP non-users, stated that “the Scene” was changing and condoms were being used less than they had been previously. These participants had different perspectives on how much condom use had decreased, but all expressed that it was significantly lower. N5 (30s, open relationship, $86 k) stated, “It was also interesting over the years that I was taking it to see the change in people’s behaviors, and the fact that when I first started taking it, in [big East Coast city], most people were still not comfortable having bareback sex. But sort of at the time that I stopped taking it, most people were very comfortable having bareback sex.”

The second theme within this category, negotiating risk, was discussed by all 13 participants and was related to the notion that PrEP use is relevant to participants’ effort to mitigate the risk of HIV infection in an environment in which social norms around sexual behavior are changing. Some (*n* = 10) participants expressed that their risk of HIV infection influenced their decision whether to use PrEP. They expressly stated that their self-perceived risk of HIV infection influenced their decision about using PrEP. U3 (20s, open relationship, $65 k), for example, stated, “My doctor kind of approached it as, “Well, like, you’re a man who has sex with men, which means you’re automatically placed in a higher risk pool for HIV and for other kinds of STDs.” Some non-users who believed they were at lower risk of HIV infection because they used condoms or exclusively topped, such as N10 (20s, single, no income declared), felt conflicted about how relevant PrEP was for them, despite their interest in it. He stated, “I mean that I’m interested in PrEP as something I would use on top of condoms [to further reduce the risk of HIV infection], and in that respect, how much in terms of safety does that add?” For participants, being on PrEP was relevant to how comfortable they felt engaging in sexual risk-taking behaviors, including more frequent CAS (*n* = 6) and more casual sex partners (*n* = 5). Both PrEP users and non-users (*n* = 4) mentioned that fear of going wild on PrEP and engaging in more sexual risk behaviors kept them or people they knew from using PrEP. N13 (30s, closed relationship, $125 k) expressly stated, “I consciously decided that I did not want to continue with PrEP because I felt that was giving me a green light to continue to be reckless.”

Participants expressed a range of views about the relevance of PrEP use to negotiating risk in several other repeating ideas (e.g., *discussing HIV status, “I could pick whether to use condoms*,*”* and *caring less and carelessness*). Some (*n* = 3) stated that once they started PrEP discussing HIV status with sexual partners was optional; their sexual partners trusted them to be HIV negative and their partners’ HIV status no longer affected them. Other participants expressed that being on PrEP made condom use optional and gave them the ability to use their judgment about when to use condoms. Most participants (*n* = 10) thought that being on PrEP made them *care less or be careless sexually*. U8 (20s, single, $60 k) stated, “I’m much more willing to put myself in situations (such as sex parties, having multiple partners at once and observing more people taking drugs), which are probably riskier, without thinking as hard as I would have in the past.”

Many participants (*n* = 10) expressed that using PrEP gave them peace of mind, reduced their worry about sex and HIV infection, and allowed them to be more comfortable during sex. Some participants experienced less worry about HIV when a PrEP user was their partner, such as U6 (30s, open marriage, $19 k): “If you know for certain that somebody else uses PrEP, you feel comfortable and you can sleep the night after. I remember how I was scared about getting HIV. Like, growing up in (religiously conservative country) and already very conservative environment, sex was already a constant sense of guilt, and after sex, was always coming the nights of terror.”

Participants (*n* = 7) talked about developing a relationship with PrEP and integrating it within their lives. For some (*n* = 5), using PrEP resulted in being more open to different situations, such as entering open relationships or having different kinds of sexual encounters, and gave them an increased sense of adventurousness. U8 (20s, single, $60 k) stated, “[PrEP] has allowed me to really explore what I’m interested in and who I’m interested in sexually, but also. .. that confidence sort of carries on to other aspects of who I am.” Other participants (*n* = 2) went a step further and stated that using PrEP not only made them feel more confident and open sexually, but also allowed them to “work through” other “issues.” For N13 (30s, closed relationship, $125 k), keeping himself protected from HIV infection in sexual encounters helped him focus on addressing his drug addiction. He stated, “I sort of felt like I was battling two different things at the same time. I was battling this drug-induced sort of sex craze and trying to not do that and also keep myself protected. And that’s where I decided that for right now, just not to worry about the sex part of it and focus more on the not the drug driven sex at this point.” Participants (*n* = 2) acknowledged that, as with any relationship, their views about PrEP have evolved whether or not they have used it. N12 (30s, closed relationship, $60 k) said, “I think, in the very beginning, it did seem like a silver bullet, in a lot of ways, and then as it’s progressed over the past five-six years, some of those some of the reality has crept in of this is a tool but it’s not a magic wand. It’s I think when it first came out it seemed like this is the solution to all the problems, and now it’s like, this is a thing that exists to help mitigate risk.”

The final theme that participants described within this category, putting yourself first, consisted of a set of ideas that represented how PrEP use allowed them to take agency over their own health. Participants (*n* = 5) expressed this view from saying that taking PrEP eliminated having to rely on someone to be truthful about their HIV status (U1, 30s, single, $10 k) to stating that they would use PrEP within a monogamous relationship (N12, 30s, closed relationship, $60 k). Experiencing a health scare also made salient that PrEP use was relevant to them for some participants (*n* = 4). U9 (30s, single, no income declared) stated, “I was always looking more into it, and then one of my friends was diagnosed with HIV and he had only been active not very much. And so, I was like, “You know? I need to get on this.” Three PrEP users stated that they were more aware of their health since they started using PrEP because they got tested for STIs more often through the routine testing required for PrEP.

#### Category 3: the continued importance of education (knowledge is power)

The third and final category, the continued importance of education or knowledge is power, refers to two themes that arose in participants’ narratives, PrEP awareness and PrEP logistics. These themes highlighted the value of being informed about PrEP and sexual health.

Many participants (*n* = 10) discussed the theme of PrEP awareness. Their experience of using PrEP was affected by their own and others’ awareness of PrEP, especially their healthcare provider. U8 (20s, single, $60 k) stated, “My allergist … just ran down the standard questions, what medications. .. I said Truvada, and then his immediate follow-up was, “What’s your viral load?” And I had to say, “Well, no. I actually don’t have HIV. I’m on it preventively. .. I’m on it for PrEP. And he just looked completely confused and bewildered and he was just, “Oh, why are you taking a medication for something when you don’t have that?” Some men stated that they sought LGBT-friendly doctors specifically due to previous experiences with doctors who were not familiar with PrEP. Participants (*n* = 6) stated that they made informed decisions about their sexual behavior, e.g., using condoms and PrEP, only once they were adequately informed about sexual health. Participants (*n* = 4) also stressed the importance of seeing PrEP public ad campaigns. The men in this study felt differently about whether research about PrEP could be trusted (*n =* 4). U1 (30s, single, $10 k) stated, “I’ll do what the professionals say not what the rumor mills say or the blogs or that sort of thing.” N4 (20s, open relationship, $85 k), on the other hand, was more skeptical and stated, “It’s pretty incredible to think that something that hasn’t really been, or to my knowledge, hasn’t been tested a lot is being pushed so hard. We don’t really know what if any long-term effects could occur from this.”

Participants discussed PrEP logistics that they considered to be the important when they decided whether to use PrEP. Participants (*n* = 11) identified logistical concerns associated with taking PrEP, such as *cost*, *effectiveness*, and *adherence*. U8 (20s, single, $60 k) stated, “If I can take this medication that I was thinking about and also not have to pay as much for healthcare? Perfect. That was sort of the reason why I decided to go on it.” Three participants described *access to PrEP* as important. N4 (20s, open relationship, $85 k) stated that he and his partner were considering taking PrEP in the future, and knowing “how easy it is to get the drug” allowed him and his partner to consider using the intervention. Participants (*n* = 6) expressed a range of views about the relevance of distinct aspects of taking a medication for PrEP, including *side effects* and *adherence*. Some PrEP non-users expressed concern about long-term side effects as a reason for not taking PrEP, while others, despite being aware of side effects, denied that side effects were a factor in their decision not to use PrEP. Participants also had different views about adherence. While some PrEP users who mentioned adherence (*n* = 5) stated that the effort of taking a medication regularly was minimal, for some PrEP non-users (*n* = 2), who had negative associations with being on medication, the idea of taking a medication regularly deterred them from taking PrEP. N10 (20s, single, no income declared) stated: “I don’t want to think about other health things and add things to my mix right now, so that’s also some of the inertia in all of this.” One participant ultimately overcame this aversion, while, for the other, it continued to be a barrier to initiating PrEP.

### Biases

The researchers consider it important to report our biases to mitigate the vulnerability of finding only what we expect [30]. The principal investigator is a white, well-educated, heterosexual woman in her 70s who has been in a monogamous relationship for 44 years. She lives in a city with a very active gay culture. She anticipated that GMSM would want to use any medication that protected them from HIV and believed such practices would bolster PrEP use. She was surprised that there were many shame issues, in and out of committed gay relationships, about using PrEP, which made the experience of using PrEP in those participants’ relational world irrelevant or negatively experienced. The co-researcher is a gay, Latinx, well-educated cisgender man. He expected that sexual pleasure, emotional intimacy, and erotic relationships would affect PrEP uptake. He was surprised that gay men shame and judge each other about the use of PrEP even within intimate relationships, and that gay men conceal the relevance of PrEP from those close to them. The third rater is a single, heterosexual, cisgender male in his 20s of Cuban, Italian, and Jewish descent. He expected PrEP use would motivate men to have more sex. He was most surprised by how pervasive both the HIV and PrEP stigma among our participants. The fourth rater is a white cisgender female who works in the business world. She expected that most men would prefer CAS and would use PrEP to protect themselves while enhancing pleasure. She was surprised by our findings of shame in and out of committed gay relationships and the irrelevant or negative experience it has on PrEP use.

## Discussion

This study sought to allow participants to discuss how PrEP affected their intimate relationships and what factors determined whether PrEP felt relevant to them and motivated them to use PrEP. The researchers hypothesized that when gay men consider using PrEP, they consider subjective and relational factors over and above their self-perceived risk. Previous studies about facilitators and barriers to PrEP uptake and adherence have identified social stigma, cost, health insurance, healthcare provider awareness, and concern about side effects as factors that allow or impede GMSM from starting PrEP [14, 31, 32]. As we discuss below, our research supports these findings.

Our research query was motivated by research that has targeted “common uncertainties around widespread PrEP implementation” [8] including risk compensation. Often, this research reduces the benefit of being on PrEP to an “objective” decrease in the risk of HIV infection [33], i.e., as straightforward and objective. Risk and safety, however, can be considered “thick concepts” [34] that are rich with idiosyncratic meaning [35]. Research addressing risk and motivations for use among GMSM who use PrEP suggests, “The meaning of an event or a phenomenon (e.g., HIV disease, [STIs]) may or may not be perceived as threatening, depending on the values held. To consider something as risky implies that something of value is at stake and is under threat” [35]. In the present study we sought to ask gay men about how risk and other factors affected their motivations about whether to use PrEP, and how risk and PrEP were relevant to their lived experience. Our final aim was to provide qualitative data about the relevance of subjective, relational, and intersubjective factors, i.e., factors beyond “objective” risk of HIV infection, in gay men’s decision to adopt or forgo PrEP. We sought to build upon the scant information we found about psychological correlates of PrEP use [33], which suggested that subjective factors (affective factors such as fear of HIV and motivational factors like pleasure and intimacy) affect GMSM’s interest in PrEP [18, 36]. Entering into committed partnerships has been observed to correlate with GMSM’s self-perceived level of risk and their discontinuation (or restarting) of PrEP [37]. The researchers came across no studies that explored what relationship, if any, PrEP use has on the establishment of committed relationships.

Thirteen gay men shared intimate details of their lives understanding they would not be compensated but that their stories would contribute to a body of research that aims to understand and help GMSM who consider using PrEP. The results of our study indicate that interpersonal factors, including group identification, committed relationships, and sexual partners, as well as changing sexual norms and specific characteristics of taking a biomedical intervention like PrEP, are all relevant to the decision about whether to use PrEP and affect how PrEP use relates to and is experienced in our participants’ lives. These findings are discussed below.

The results of our study indicate that, for the participants of our study, there is a specific sense of “what it’s like to be someone who is on PrEP,” a category described by participants that comprised how social and interpersonal factors, including group identification, committed relationships, and sexual partners, have a significant bearing on the motivations to use PrEP and on the sense of whether PrEP is relevant for individuals.

The notion of the inextricable mutuality between the individual’s identity processes and the relational matrix has been referred to, among other things, as the “holon” (the “whole”) by structuralist systemic thinkers such as Minuchin [38]. Our participants’ narratives evoked a similar idea when they described how the social environment determined what they thought about using PrEP. PrEP-using participants and some non-users who previously used it stated that talking to friends about PrEP and hearing friends talk about using PrEP allowed them to take PrEP more seriously and to consider using it themselves. Previous findings have identified similar relationships between sexual and social relationships and the likelihood of using PrEP [39]. Participants talked about how their friends viewed PrEP differently than how they talked about the “gay world’s” attitude toward PrEP. One participant who previously used PrEP stated that being on PrEP is “almost the norm within the gay world” (N4, 20s, open relationship, $85 k). Concern about the gay community motivated participants to use PrEP to be “responsible” by “doing their part” to stop the spread of HIV. Many participants expressed that feeling normal and being part of a community were ideas that were embedded in their experience of PrEP use.

Some participants who considered using PrEP ultimately felt that PrEP was less relevant for them, and the reasons for this varied. Some of these men denied that their friends talked about being on PrEP. Some participants stated that sex stigma affected their decision about PrEP use. Consistent with Grace and his team’s findings that stigma is experienced in multiple ways among PrEP users [18, 40], the men in our study experienced stigma in many ways, including concern about HIV, negative bias against PrEP use, and negative judgments about casual sex, barebacking, or non-monogamy. The significance of HIV among GMSM has been described in previous research [41]. Most if not all participants also recognized that HIV stigma, the fear that people living with HIV “are sick” (U1, 30s, single, $10 k) and contagious, continues to be prevalent, if less intense than it was in the past. Some men who felt concerned about their risk of HIV infection and were aware of the stigma against HIV positive men felt motivated to start PrEP. Men in the present study also differentiated HIV from other STIs, describing HIV as something that antibiotics cannot “fix” (U3, 20s, open relationship, $65 k), that is, incurable and permanent, which aligns with previous research findings (e.g., [42]).

Stigmatizing views about PrEP use influenced how relevant PrEP felt for our individuals and whether they decided to start PrEP. Participants who experienced stigma because they were on PrEP described being stereotyped as being promiscuous and sexually irresponsible, “you must have no value, basically, like a cum rag. .. just pieces of ass or just people to fuck” (U1, 30s, single, $10 k). Previous research has revealed similar findings [11, 18, 40, 43]. Thomann and colleagues found that stigma against PrEP use appears to be decreasing [44], which fits our participants’ narrative of PrEP use being increasingly acceptable. However, for our participants, PrEP use continued to carry negative associations when paired with specific behaviors (e.g., barebacking with casual sex partners, bottoming). This mirrors findings in recent research indicating that GMSM have a range of evolving attitudes towards PrEP that may be mediated by attitudes towards specific sex acts [45–47].

For the men in our study, using PrEP felt relevant to their sexual relationships both within casual sexual encounters and in the context of committed partnerships, and the effect of using PrEP on these relationships varied considerably. Some men expressed a decreased need to trust their sexual partners. Many participants expressed uncertainty about whether using PrEP felt “right” in monogamous relationships because they felt a tension between trusting their partners, and wanting to communicate this to them, while wanting personal peace of mind from taking care of their own health. This was a novel finding, as previous research has predominantly focused on the discontinuation and reinitiating of PrEP when individuals form or dissolve monogamous relationship contracts, respectively, and has usually attributed these phenomena to changes in individuals’ self-perceived level of risk [37]. Some PrEP non-users in our study described experiences reminiscent of Carlo Hojilla’s team’s findings. For other participants, using PrEP within closed relationships involved considering what their partner’s perception would be if they chose to stay on PrEP. Participants’ views about PrEP use within open relationships was more consistent. Participants whose relationship agreements incorporated the use of PrEP believed both couple members were taking measures to protect each other’s health. Some men in our sample also expressed differing views on whether using PrEP influenced their interest in establishing committed partnerships. Our participants surmised that PrEP might decrease the desire to be in a committed partnership by enabling some participants to feel comfortable having more sex or barebacking, both of which might have been incentives to be in monogamous relationships previously. For many participants, PrEP use created an opportunity to negotiate their desire for sexual intimacy with their desire for self-preservation consciously. These findings support recent findings about the multitude of ways in which PrEP use can be facilitated or complicated within primary sexual relationships [28, 48–52].

Participants spoke specifically about changing sexual norms in their communities.

Participants in this study described an evolving social and sexual environment in which safe sex fatigue, experienced as dissatisfaction with the norm and frustration with the constraints placed by condom use, was punctuated by PrEP’s development and widespread uptake.

Many participants expressed dissatisfaction with condoms because of their unreliability, the physical and psychological strictures of having to put on a condom, and the decreased sensation and loss of pleasure from their use. Dissatisfaction with condoms has appeared in the extant literature as a possible factor associated with PrEP adoption [33]. Some participants acknowledged that using PrEP felt relevant because of the general decrease in the prevalence of condom use. Our participants reported that their sexual partners often did not use condoms and that they experienced pressure to engage in CAS. Previous literature has suggested that there are greater rates of CAS among GMSM in the past two decades [28]. In partial contrast with previous findings, the men in our study denied that wanting to bareback more themselves was a reason for starting PrEP. They did say, however, that they wanted to start PrEP in part because others wanted to bareback more.

Some participants said that PrEP allowed them to have more spontaneous and pleasurable sexual experiences. For them, sex became liberating and comfortable, and they were no longer beset by worry about possible HIV infection. Previous research has described similar experiences about decrease in anxiety and worry about HIV infection among individuals who use PrEP [33, 37]. Participants conveyed a greater sense of satisfaction in their sexual encounters. This is surprising given that previous research about the effect of PrEP on sexual satisfaction did not find evidence of a relationship between PrEP use and sexual satisfaction [33]. Whitfield and his team found that being in a relationship and having more CAS experiences were associated with greater sexual satisfaction, but PrEP use itself was not predictive of this increase. Whitfield’s team conjectured that, “Taken together with the decrease in sexual anxiety, it is possible that sexual satisfaction for these participants is linked not simply to a fear of HIV acquisition but to a deeper sense of contentment not predicted by PrEP use” [33].

Participants’ narratives seemed to reveal a dynamic process of compromise between risk and sexual satisfaction. Some participants who used PrEP considered themselves at high risk of HIV infection prior to starting PrEP. Most PrEP users related engaging in sexual risk behaviors and associated changes in their sexual behavior to being on PrEP. Some PrEP users had riskier sex by barebacking, bottoming, or having group sex, while others reported having more sex or more sexual partners. Our findings are consistent with previous research indicating that some GMSM who start using PrEP report engaging in more sexual risk behaviors as a result [8]. The idea of risk compensation was perceived as liberating and also as scary, potentially dangerous, and reckless by some of our participants. Some PrEP non-users wanted to avoid using PrEP out of fear that they might start to engage in riskier behavior. Such experiences of fearing one’s own disinhibition as a reason for forgoing PrEP use was not one the researchers came across in their review of the literature. Previous studies about PrEP have suggested that some GMSM anticipate that they would engage in more CAS on PrEP [35]. However, in these studies anticipated sexual disinhibition was not a reason for not taking PrEP, as was the case in the current study. Some PrEP users in our study talked about being less cautious and putting themselves at risk without thinking about it, an idea also raised by PrEP non-users. For some participants, it became difficult to draw the line about what was too risky when barebacking was considered to be safe.

The researchers wonder whether participants’ process of figuring out their limits might be related to other phenomena they described. Participants expressed that PrEP opened them up to new experiences, allowed them to work through issues, and enabled them more fully to explore their sexuality and become more confident, both sexually and as a whole. Some described increased ability to make their own decisions about how they engage sexually, from choosing whether to ask about HIV status or whether to wear a condom. For participants who described starting PrEP or considering starting PrEP because of a health scare, taking PrEP represents self-care. PrEP’s effect on sexual esteem, or the “positive feelings they have about their sexual activity and ability to deal effectively with the sexual aspects of themselves” [33] has not yet been well studied [33].

Some participants’ attitudes about PrEP itself evolved as they integrated PrEP into their lives and taking it became the new normal. The researchers are unaware of previous studies that have investigated how PrEP users’ attitudes about PrEP change over time.

Moreover, participants also discussed the relevance of different characteristics of using a biomedical intervention like PrEP on their motivations to use PrEP and on their sense of whether PrEP was relevant to them. Participants frequently mentioned the importance of education and awareness about PrEP. Many participants stated that it was important to them that physicians be aware of PrEP and that they have experience prescribing it. There is ample evidence that many GMSM continue to feel stigmatized within the context of their relationship with their primary care providers [53]. Additionally, physicians appear to have implicit biases against individuals who engage in sexual risk behaviors, which compromises doctors’ willingness to prescribe PrEP [54]. Participants in our study often attributed their own willingness or resistance to being on PrEP to familiarity with PrEP or lack thereof. Ads about PrEP, which numerous users and non-users reported having seen, increased PrEP visibility and made these men consider PrEP more relevant for them. Participants’ views ranged from “It’s almost the norm to use PrEP in the gay world” (N4, 20s, open relationship, $85 k) to “I think people just don’t know it’s there” (U3, 20s, open relationship, $65 k). This may suggest that some participants in this and previous studies identify with being privileged and having more knowledge than some of their peers. Indeed, as one PrEP-using participant observed, “I say all these things coming from what I recognize is a position of extreme relative privilege. .. I’m a cis-gender white male living in [the heart of a big city with] health insurance, a job that affords me that health insurance, and I can, barring some catastrophe, usually afford to pay my medical bills, which is not necessarily the case for this entire community, um, you know, particularly our trans brothers and sisters, people of any color” (U3, 20s, open relationship, $65 k).

Most participants identified logistical factors such as cost, health insurance, and accessibility as important, and they considered these factors when deciding whether to start PrEP. This trend has been robustly observed in previous research [17].

Some participants who knew more about PrEP declared that PrEP felt more relevant for them. These participants appeared to be less deterred by concerns such as resistance to taking a medication, worry about side effects or the effort of adhering to a drug regimen. Trust in medical research and health professionals also influenced participants’ interest in PrEP. Participants who knew less about PrEP or felt less certain about the data on PrEP were less certain that PrEP was relevant for them. This, too, has been supported in previous literature in which participants’ skepticism of medical professionals is a reason why they do not seek talk to their providers about PrEP [55].

Some men in our study conjectured that increasing campaigns to promote PrEP education, particularly ones not only targeting gay men, might help increase uptake. The researchers of this study agree. Participants speculated that insufficient education about PrEP likely results in lack of awareness about PrEP and contributes to PrEP’s low uptake. This conjecture is only partially borne out by the data, as rates of PrEP awareness are close to 90% [2], and it is worth revisiting to clarify why even relatively privileged and well educated GMSM continue to believe that awareness is low among their communities.

### Limitations

Recruitment was challenging. This difficulty may have arisen due to the topic, which required that subjects describe their sexual behaviors and discuss their consideration of PrEP or due to a lack of established relationships between the researchers and local community centers. The participants in this study were largely a homogenous group primarily consisting of young, White, affluent, highly educated, gay-identifying HIV-negative cisgender men. Participants were only asked if they were “sexually active” generally, and they were not asked to state more clearly what recent sexual activity or sexual risk behaviors were. These data were elicited over the course of the interview for many but not all participants. None of the men were in serodiscordant relationships. Although we reached saturation, our sample size was more homogeneous than was initially our goal. Considering the difficulty recruiting a random sample of participants and with the inherent bias in qualitative research (namely, that of the researchers’ own biases), the main limitation to this study is the generalizability of the findings.

Additionally, despite the study’s inclusion of a comparable number of PrEP users and non-users, a limitation of these findings was that the number of individuals included who were not and had never been on PrEP was small. We could have approached recruitment and the research questions differently to incorporate the views of men who had never used PrEP. Thus, the researchers note that in this study we mostly saw why GMSM might use PrEP but not so much why they do not or would not use it. It is also possible that reasons for not using PrEP are, in fact, largely due to external constraints on access to PrEP.

### Future research

The results of this study have inspired us, the researchers, to consider areas of future study that might enrich and clarify the current literature. First, and foremost, future researchers investigating PrEP use might diversify and expand the sample of participants involved. Ideally, the sample recruited would include participants of varying age, level of education, SES, race, and geographic regions. The researchers of this study believe that future research about the casual and committed sexual partners of individuals on PrEP might also proffer valuable insights that complement the experience of PrEP users themselves. Finally, we also believe that trans women and substance using GMSM represent two additional vulnerable populations in the U.S. burdened by high HIV rates, and their experiences should also be targeted by future PrEP research.

Comparing the experiences of GMSM of different backgrounds would shed light on their views of related phenomena, such as barebacking, PCNM, HIV, casual sex, Grindr and/or other location-based hookup and dating apps.

Additionally, given that PrEP is a biomedical intervention, future research might consider the experiences of both physicians and mental health practitioners working with GMSM for whom PrEP might be useful. For physicians, education about various reasons why GMSM might seek PrEP might serve to mitigate their bias against patients whom they see as sensation-seeking or high risk. More education about the diverse experiences that could benefit from PrEP use might allow physicians to recommend PrEP for people who do not fit the “standard” PrEP user profile (e.g., single, sexually active GMSM). Fuller awareness about the experiences of GMSM who have considered PrEP might help mental health workers suggest PrEP to patients they think will benefit from it and to help them anticipate difficulties and benefits that are more relevant to them. Greater sensitivity to differences among PrEP users might reduce sex-negative bias and enhance clinicians’ ability to empathize with patients whose behavior they consider risky.

Finally, should our findings be found to generalize to larger samples, they may inform future PrEP advertisements and help them appeal to a more diverse population with a wider array of experiences. Better ads might allude to experiences that appeal to higher risk groups (such as young GMSM or GMSM of color). We lacked such participants, but if our findings apply to those populations, advertising might shift to emphasizing that taking PrEP is socially responsible. Future ads might try to normalize PrEP use (rather than merely highlighting its risk-reducing effectiveness) such that even its use within committed partnerships is seen as responsible preventive healthcare. Future attempts to increase uptake should also incorporate education about HIV and other STIs in a way that is not stigmatizing but nonetheless conveys their seriousness.

The feasibility of adopting these and other measures, however, depends on the availability of relevant, current research. We, the researchers of this study, firmly believe in the power of education and information, and hope that, by giving greater attention to the experiences of GMSM and their use of PrEP, future research will be poised to help deliver meaningful messages to others that safely enrich their individual lives and their relationships.

## Conclusions

In this study, the researchers spoke with 13 GMSM who shared their experiences of PrEP, how they gauged its relevance to them, and how their lives have changed since this decision. To address gaps in the literature and develop greater insight about PrEP’s meaning in the cultural context of gay men specifically, we asked both gay men who used PrEP and those who contemplated PrEP but did not initiate it about what factors were relevant when they decided whether to use and to the experiences undergirding these decisions. We anticipated many of our findings, and were surprised and intrigued by others.

Many of the phenomena described by these men have been identified in previous research: self-perceived risk [56], PrEP acceptability [17, 24], access and affordability of PrEP and LGBT-aware healthcare [17], dissatisfaction with condoms [33], concern about taking medication and its side effects [17], risk compensation [8], decreased anxiety about HIV infection during sex [26], and HIV- and PrEP-related stigma [18, 40]. It is both heartening and perplexing to see that many of our findings have been observed in previous research, as described above, given the shortcomings in reaching at-risk GMSM and increasing PrEP uptake [2].

Our participants also elaborated on themes that have not been researched in depth and that may serve as areas for future research. Participants identified health scares as potential catalysts for starting PrEP, and many identified as sexually and socially responsible individuals who want to help stop the spread of HIV. For many participants sex became riskier. Some experienced the changes as liberating; they described feeling more sexually comfortable and able to engage in sex more freely. Others, in contrast, were taken aback by their disinhibition. They described that they feared losing control of their sexual agency and going wild—a new idea that emerged from our participants’ narratives. Some participants shared ways PrEP use may affect them, including promoting greater agency, allowing them to become more sexually open, and enabling them to develop confidence and work through issues. We speculate that satisfaction may increase when one’s sexual way of life feels increasingly less singled out as having greater health and stigma cost than that of the ways of others. PrEP is not only a pill that GMSM are told they need, but also a method of self-care, freedom, agency, and contentment.

Moreover, many of these men described their experiences not as single, independent agents but rather as members of committed partnerships. Some participants felt PrEP affected their interest in committed relationships. Some grappled with whether PrEP use was right within their committed relationship, whether open or closed, and about the protection and trust it conferred on them as individuals and as a couple. Some spoke of conflict deciding whether to use PrEP in monogamous relationships due to what this could signify to their partners, fearing it might foster distrust. Some men also shared that they and their partners were in ongoing dialogue about their sexual contracts about whether to incorporate PrEP, given their combined level of risk. These ideas have not been deeply explored in prior research. We suggest that researchers, in failing to “ask about issues of love, commitment, and affiliation, and, instead, focusing on sexual aspects of these relationships” [57], have largely ignored consequential ways in which PrEP use is a part of GMSM’s lived experiences. Large scale, future research might test the generalizability of these ideas.

A grounded theory narrative, not to be overgeneralized to anyone beyond our sample, might be as follows: GMSM who consider using PrEP have many points of entry into its use and of decision making. If and when their culture is one where friends, doctors, and romantic partners, as well as public advertisements seem to support PrEP’s use, the men may go ahead and try to get an affordable prescription. If, however, any one of those influences is negative, mistrusting, or shaming, that influence may be felt strongly enough to dissuade choosing to use PrEP. Another choice point can come when the men are exposed to health scares, either in themselves, in friends or in partners. The scares may trigger a sense of needing to take better care of themselves. Some men, however, will not take PrEP because they believe that they will have worse problems on which to focus their energies or that they will get into more health risk situations if they were to take PrEP. This bias is an implicit leaning shared by some health care workers who hold back prescribing PrEP. The participants may simply not want to entrust themselves to PrEP when they cannot trust the science. Commitments, either to one’s partner or to the gay culture as a whole, also affect men’s choices. Some go one way, feeling that all will be safer if they use PrEP, and some the opposite, that is, they fear they will be mistrusted for any commitment promises made in relationships. The men who decided not to use PrEP know it is always out there. Some men who decided to use PrEP seem to feel an added sense of belonging and peace of mind. Others felt more sexually satisfied and more secure about being able to take care of themselves and those they care about, from their partners to their community. It is notable that the men had many dissatisfactions with condoms as a prophylactic; however, the men who used PrEP did not base their decisions about using PrEP solely on their dissatisfactions with condoms. Our participants expressed that interpersonal and social factors, the primary romantic relationship issues around the perception of trustworthiness and the group norms, the sense of acceptance in society (e.g., ads and clinics) also greatly shaped their attitudes towards PrEP and their experiences of it.

Our participants feel a social responsibility to themselves and to the community at large. They seem predominantly to welcome the increased pleasure and peace of mind and perhaps even feel less stigmatized since they may take their sexual pleasures with greater ease than before PrEP. They respect both monogamous relationships and consensual non-monogamous ones; the current authors hope that others may return their respectful attitudes to them. We hope that our conclusions aid that wish’s fulfillment. Some of these men may appear to outside observers to have become careless about their health in risking HIV infection. However, they seem to have made thought-through choices given the treatable nature of those illnesses. Finally, we think the persistent difficulties these men have in finding educated health practitioners requires a major focus among health care educators and the curricula they provide.

## Supplementary Information

**Additional file 1.** Demographic Questionnaire. Questions posed to participants at the outset of the study about relevant demographic information

**Additional file 2.** Semi-structured Interview. Prompt and potential prompts developed to organize clinical interview

**Additional file 3: Tables 1a and 1b**. Participant Demographic Data Results (.DOC) General and individual participant demographic data

**Additional file 4: Table 2**. Categories, Themes, and Repeating Ideas (.DOC) Summary of categories, themes, and repeating ideas

## Data Availability

The datasets generated and/or analyzed during the current study cannot be made publicly available as they were purged following the conclusion of the study, per the IRB.

## Abbreviations

CAS: Condomless anal sex
FDA: U.S. Food and Drug Administration
GMSM: Gay and other men who have sex with men
HIV: Human immunodeficiency virus
iPrEx: Preexposure prophylaxis initiative (from Spanish original, Iniciativa Profilaxis Pre-Exposición)
PCNM: Partnered consensual nonmonogamy
PrEP: Pre-exposure prophylaxis
RI: Repeating idea
STI: Sexually transmitted infection
