# Supplementary material for: The relevance of pre-exposure prophylaxis in gay men’s lives and their motivations to use it: a qualitative study
Source: BMC Public Health. 2021 Oct 9;21:1829. doi: 10.1186/s12889-021-11863-w (PMC8502319; doi:10.1186/s12889-021-11863-w)
Supplement: Supplementary file 1 — Additional file 1. Demographic Questionnaire. Questions posed to participants at the outset of the study about relevant demographic information [file 12889_2021_11863_MOESM1_ESM.docx]

**Demographic Data Sheet**

**Directions: Please respond to each question as best you can.**

Sex: Gender:

Age: Race/ethnicity:

Highest education level (e.g., some high school, college):

HIV status:

Occupation: Estimated annual income:

Sexual orientation:

Have you been sexually active in the past six months?

Are you currently in a committed relationship?

- 1. How long have you been in this relationship?
  2. Are you and your partner sexually monogamous or non-monogamous?
     1. Are you and your partner in a consensually non-monogamous (“open”) relationship?
  3. What is your partner’s HIV status?
