# Supplementary material for: The relevance of pre-exposure prophylaxis in gay men’s lives and their motivations to use it: a qualitative study
Source: BMC Public Health. 2021 Oct 9;21:1829. doi: 10.1186/s12889-021-11863-w (PMC8502319; doi:10.1186/s12889-021-11863-w)
Supplement: Supplementary file 2 — Additional file 2. Semi-structured Interview. Prompt and potential prompts developed to organize clinical interview [file 12889_2021_11863_MOESM2_ESM.docx]

**Semi-Structured Interview**

**Prompt:**

We’re interested in knowing your thoughts and feelings about PrEP, and whether you use it or you don’t use it. We’re interested in the relationship of its use (or not) to your intimate sexual relationships and sex practices. Could you please tell us?

**Potential Prompts:**

1. How did you learn about PrEP?
2. What was going on in your life when you decided to take PrEP, and how did you decide whether to take it?
3. How do you think being on PrEP has affected you?
4. Does being on PrEP affect your willingness to be in a committed relationship?
5. Does being on PrEP affect your willingness to be in an open relationship?
6. Does being on PrEP affect your willingness to be in a relationship with someone who is poz (HIV+)?

**PrEP Use**

1. Have you considered using PrEP?
2. Are you currently on PrEP?
3. How often do you take your PrEP medication?
4. Have you experienced side effects while taking PrEP?
5. What is your HIV status?
   1. When were you last tested?
