# Supplementary material for: The relevance of pre-exposure prophylaxis in gay men’s lives and their motivations to use it: a qualitative study
Source: BMC Public Health. 2021 Oct 9;21:1829. doi: 10.1186/s12889-021-11863-w (PMC8502319; doi:10.1186/s12889-021-11863-w)
Supplement: Supplementary file 3 — Additional file 3: Tables 1a and 1b. Participant Demographic Data Results (.DOC) General and individual participant demographic data [file 12889_2021_11863_MOESM3_ESM.docx]

**Table 1a: Participant Demographic Data, General (N = 13)**

| **Age** | **Number** | **Percent** |
| --- | --- | --- |
| 18-24 | 2 | 15.4 |
| 25-34 | 9 | 69.2 |
| 35-44 | 2 | 15.4 |
| **Racial identification** |  |  |
| White/Caucasian | 12 | 92.3 |
| Biracial | 1 | 7.7 |
| **Relationship status** |  |  |
| Single | 7 | 53.8 |
| Partnered, monogamous | 1 | 7.7 |
| Partnered, “monogamish” | 2 | 15.4 |
| Partnered, open | 1 | 7.7 |
| Married, monogamous | 1 | 7.7 |
| Married, open | 1 | 7.7 |
| **Highest level of education** |  |  |
| College/Bachelor’s | 5 | 38.5 |
| Graduate/Doctoral | 4 | 30.8 |
| Unknown | 4 | 30.8 |
| **Occupation** | **Number** | **Percent** |
| Service Industry | 4 | 30.8 |
| Science, Technology, Math | 4 | 30.8 |
| Applied and Fine Arts | 3 | 23.1 |
| Nonprofit | 2 | 15.4 |

**Table 1b. Participant Demographic Data, Individual**

| **Participant** | **Age** | **Race/Ethnicity** | **Education** | **Income  ($/yr.)** | **Relationship status** | **HIV status** | **Time on PrEP (mo.)** |
| --- | --- | --- | --- | --- | --- | --- | --- |
| U1 | 30s | Latino/White | Bachelor’s | 10k | Single | Negative | 24 |
| U2 | 30s | White | - | 35k | Single | Negative | 2 |
| U3 | 20s | White | Bachelor’s | 65k | Open | Negative | 42 |
| N4 | 20s | White | Bachelor’s | 85k | Open | Negative | N/a |
| N5 | 30s | White | Graduate | 86k | Open | Negative | N/a |
| U6 | 30s | White | Graduate | 19k | Open | Negative | 48 |
| U7 | 30s | White | Bachelor’s | 140k | Single | Negative | 12 |
| U8 | 20s | White | Bachelor’s | 60k | Single | Negative | 21 |
| U9 | 30s | White | Bachelor’s | - | Single | Negative | 48 |
| N10 | 20s | White | Bachelor’s | - | Single | Negative | N/a |
| N11 | 20s | White | Bachelor’s | 45k | Single | Negative | N/a |
| N12 | 30s | White | Graduate | 60k | Closed | Negative | N/a |
| N13 | 30s | White | Bachelor’s | 125k | Closed | Negative | N/a |
