# Supplementary material for: The relevance of pre-exposure prophylaxis in gay men’s lives and their motivations to use it: a qualitative study
Source: BMC Public Health. 2021 Oct 9;21:1829. doi: 10.1186/s12889-021-11863-w (PMC8502319; doi:10.1186/s12889-021-11863-w)
Supplement: Supplementary file 4 — Additional file 4: Table 2. Categories, Themes, and Repeating Ideas (.DOC) Summary of categories, themes, and repeating ideas [file 12889_2021_11863_MOESM4_ESM.docx]

**Table 2: Categories, Themes, and Repeating Ideas^^[[1]](#footnote-1)^^[[2]](#footnote-2)^^**

| **Repeating Idea** | **Number of Participants** | | **%** | **Participants** |
| --- | --- | --- | --- | --- |
| **Category 1:**  What’s It’s Like to Be Someone Who’s on PrEP | | | | |
| **Theme 1: Belongingness and social acceptability** | | | | |
| *Talking to friends* | 5 | | 38% | U2, N4, N5, U8, N10 |
| *Social acceptability of PrEP in the Big City* | 5 | | 38% | U2, N4, U7, U8, U9 |
| *Social responsibility* | 4 | | 31% | U6, U7, U9, N11 |
| **Theme 2: Fear and stigma associated with sex** | | | | |
| *Historical fear and current HIV stigma* | 10 | | 77% | U1, U2, U3, N4, N5, U6, U7, U8, N10, N12 |
| *PrEP stigma* | 7 | | 54% | U1, U3, U6, U7, U8, N11, N13 |
| *Decrease in HIV stigma* | 6 | | 46% | N4, N5, U6, U7, U8, N12 |
| *HIV is worse than other STIs* | 5 | | 38% | U3, N5, U8, N12, N13 |
| **Theme 3: Trust and sexual relationships** | | | | |
| *PrEP within a monogamous relationship* | | 11 | 85% | U1, U2, U3, N4, N5, U6, U7, U9, N10, N12, N13 |
| *Trust in casual sexual partners* | | 6 | 46% | U1, U2, U3, U8, N10, N12 |
| *PrEP within open relationships* | | 5 | 38% | U3, N5, U6, U8, N13 |
| *Interest in being in a committed relationship* | | 4 | 31% | N5, U7, U8, U9 |
| **Category 2:**  An Environment of Changing Sexual Norms | | | | |
| **Theme 4: Dissatisfaction with condoms** | | | | |
| *Spontaneity* | 7 | | 54% | U1, U3, N5, U6, U7, U8, U9 |
| *Condom dis-pleasure and bareback pleasure* | 8 | | 62% | U1, U2, U3, N4, N5, U7, U8, N12 |
| *People don’t use condoms* | 5 | | 38% | U2, N5, U7, U8, N10 |
| *Condoms break* | 3 | | 23% | U2, U6, U9 |
| **Theme 5: Negotiating risk** | | | | |
| *Self-perceived risk of HIV infection* | 10 | | 77% | U1, U3, N4, N5, U8, U9, N10, N11, N12, N13 |
| *Changes in condom use* | 8 | | 62% | U1, U2, U3, N4, N5, U7, U8, U9 |
| *More sex, more partners* | 5 | | 38% | U3, N5, U6, U7, U9 |
| *Fear of going wild* | 4 | | 31% | U1, U8, N10, N13 |
| *Discussing HIV status* | 3 | | 23% | U1, U3, N4 |
| *“I could pick whether to use condoms”* | 3 | | 23% | N4, U6, N11 |
| *Caring less and carelessness* | 10 | | 77% | U1, U2, U3, N4, N5, U6, U7, U8, N10, N13 |
| **Theme 6: Peace of mind** | | | | |
| *Sex became liberating and comfortable* | 8 | | 62% | U1, U2, U3, N4, N5, U6, U7, U8 |
| *Less worry about HIV infection* | 8 | | 62% | U2, U3, N5, U6, U7, U8, U9, N12 |
| **Theme 7: Developing a relationship with PrEP** | | | | |
| *Openness to new experiences* | 5 | | 38% | U2, N5, U6, U8, N13 |
| *Working through my “issues”* | 2 | | 15% | U8, N13 |
| *My attitude about PrEP changed* | 2 | | 15% | U8, N12 |
| **Theme 8: Putting yourself first** | | | | |
| *Self-care and agency* | 5 | | 38% | U1, U2, U3, U8, N10, N12 |
| *Starting after a health scare* | 4 | | 31% | U7, U9, N10, N13 |
| *I got tested more* | 3 | | 23% | U3, U6, U8 |
| **Category 3:**  Continued importance of education (Knowledge is power) | | | | |
| **Theme 9: PrEP awareness** | | | | |
| *Importance of provider knowledge/experience* | 7 | | 54% | U3, U6, U8, U9, N11, N12, N13 |
| *Education* | 6 | | 46% | U1, U3, U6, U7, U8, N11 |
| *Advertisements* | 4 | | 31% | U1, U3, N4, U6 |
| *Trust in the medical system* | 2 | | 15% | U1, N4, U6, N11 |
| **Theme 10: PrEP logistics** | | | | |
| *Health insurance and cost* | 9 | | 69% | U1, U3, N4, U7, U8, U9, N11, N12, N13 |
| *Concern about side effects* | 6 | | 46% | U1, U2, N4, U8, N11, N13 |
| *Adherence is easy, and it makes things easy* | 5 | | 38% | U1, U3, U7, N10, N12 |
| *Accessibility* | 3 | | 23% | U2, N4, N11 |
| *Medication aversion* | 2 | | 15% | U2, 12 |

1. Underlined text refers to categories, bolded text refers to themes, and italicized text refers to repeating ideas. [↑](#footnote-ref-1)
2. U designates participants who use PrEP, and N those who do not use PrEP. [↑](#footnote-ref-2)
